# Expensive Laboratories at Hospitals

**Published:** 1905-04-01

**Authors:** 


### EXPENSIVE LABORATORIES AT HOSPITALS.

For over thirty years I was pharmacist at two of our large London Hospitals, where I had ample opportunities of studying the subject referred to in a paper read before the Hospitals Association on March 3rd by Dr. D. J. Mackintosh, medical superintendent, Western Infirmary, Glasgow, and in many points I am quite in agreement with him. My instincts were always in favour of making my own preparations up to a certain point, that point being so long as it did not involve the purchase and employment of costly machinery and specialised expensive labour. My early experience provided me with a most valuable lesson. An expensive laboratory was built costing over £1,000, and it never earned the institution one penny, as its maintenance was far more costly than its earning power. Probably if the machinery could have been kept working from morning to night it would have paid its way; but such was not the case, nor was it possible to so employ them. I do not hesitate to say that a plant costing £50 would have sufficed for all the work we did. My statement is confirmed by the fact that on taking up another appointment, where the plant was of a most simple character, and no laboratory existed, we actually made a larger variety of galenicals than were being made at the larger laboratory. I am, etc., Ph.C., F.C.S.

